# Structure and reactivity in neutral organic electron donors derived from 4-dimethylaminopyridine

**DOI:** 10.3762/bjoc.6.73

**Published:** 2010-07-05

**Authors:** Jean Garnier, Alan R Kennedy, Leonard E A Berlouis, Andrew T Turner, John A Murphy

**Affiliations:** 1 WestCHEM, Department of Pure and Applied Chemistry, University of Strathclyde, 295 Cathedral Street, Glasgow G1 1XL, U.K.; 2 PR&D Laboratory Building, AstraZeneca, Silk Road Business Park, Charterway, Macclesfield SK10 2NA, United Kingdom

**Keywords:** dication, 4-DMAP, electron donor, electron transfer, radical cation, redox, reduction

## Abstract

The effects on the redox properties of modifying the molecular skeleton of neutral bis-2-(4-dimethylamino)pyridinylidene electron donors, derived from 4-dimethylaminopyridine (4-DMAP), have been explored, by varying two parameters: (i) the length of a polymethylene chain linking the two pyridine-derived rings and (ii) the nature of the nitrogen substituents on the 4 and 4′ positions of the precursor pyridines. Restricting the bridge length to two methylene units significantly altered the redox profile, while changes in the nitrogen-substituents at the 4 and 4′ positions led to only slight changes in the redox potentials.

## Introduction

**Figure 1:**
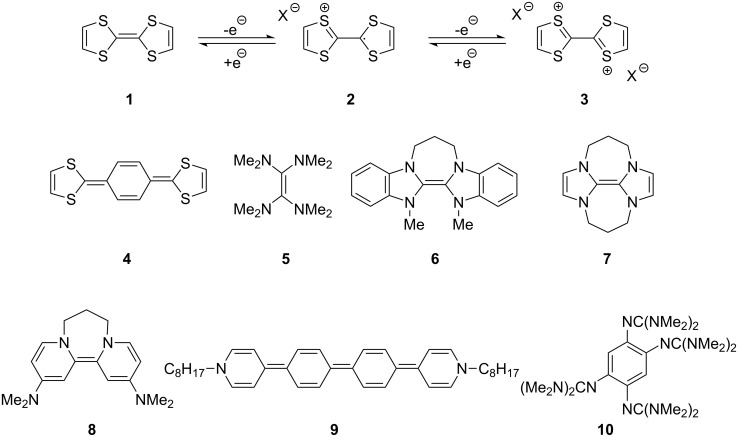
Neutral organic electron donors **1** and **4**–**10**.

Neutral organic compounds **1** and **4**–**10** (Figure 1) have attracted considerable attention as ground-state electron donors [1–38], and many are now being employed as reagents in organic transformations. Such a range of reagents with different redox potentials leads to the expectation of considerable selectivity in their reductions of organic substrates, and evidence is steadily accumulating to support this. Tetrathiafulvalene (TTF, **1**, *E*^1^_1/2_ = 0.37 V; *E*^2^_1/2_ = 0.67 V in DCM vs SCE) [1], one of the weakest of these donors, reduces arenediazonium salts to aryl radicals [2–12], but is not strong enough to react with alkyl and aryl halides. The driving force for its oxidation is the attainment of some degree of aromaticity in the formation of its radical cation salt **2** on the loss of one electron, and full aromaticity in its dication salt **3** on loss of two electrons, as well as the stabilization of both the positive charge and radicals by the lone pairs on the sulfur atoms. The effect of aromatic stabilization is enhanced in the extended analogue **4**; however, unlike TTF, this compound affords only an irreversible oxidation *E*_p_ = −0.14 V in MeCN (assuming that the reported value is measured relative to SHE, that would correspond to −0.38 V vs SCE) [13]. Tetrakis-dimethylaminoethene (TDAE, **5**: *E*^1^_1/2_ = −0.78 V; *E*^2^_1/2_ = −0.61 V vs SCE in MeCN) is a stronger reducing agent and converts electron-deficient alkyl bromides to the corresponding anions [14–17] and notably the iodide CF_3_–I to trifluoromethyl anion, ^−^CF_3_, [15] but is not powerful enough to react with aryl halides. Despite not experiencing any aromatic stabilization on oxidation, the molecule is such a good donor as a result of the ability of the nitrogen atoms in **5** to stabilize both the positive charge and an unpaired electron upon oxidation; this stabilization is greater than is afforded by sulfur in TTF.

Benzimidazole-derived donor **6** (*E*^1^_1/2_ = −0.82 V; *E*^2^_1/2_ = −0.76 V vs SCE in DMF) [18–20], combines the stabilization of positive charge and of an unpaired electron provided by four nitrogens, with aromatic stabilization in its oxidised forms. This exceptional donor has the power to reduce aryl iodides (*E*^0^ = −2.2 V) to aryl radicals, but not to aryl anions [21]. This is paradoxical in view of the standard potential of the second step; *E*^0^ = 0.05 V vs SCE in MeCN for the conversion of an aryl radical to an aryl anion [39]. Whatever about the standard potentials, in practice, the formation of aryl anions is only observed when the electron donor has *E*_1/2_ = −1 V or is more negative [40]. In line with this, both the imidazole-derived donor **7** (*E*_1/2_ = −1.20 V vs SCE in DMF) [22–25] and the 4-dimethylaminopyridine (4-DMAP)-derived donor **8** [*E*_1/2_ (DMF) = −1.69 V vs Fc/Fc^+^] [26–29], which would equate to −1.24 V vs SCE [*E* (DMF)_Fc/Fc+_ = 0.45 V vs SCE] [41] react with aryl iodides to afford aryl anions. As an indication of their enhanced donor properties, these two donors can also cleave appropriate arenesulfonamides [25], aryl alkyl sulfones [25–26], Weinreb amides [28] and acyloin derivatives [29]. They are also prone to transfer two electrons rather than one, with the cyclic voltammogram (c.v.) of **8** showing a single 2-electron reversible redox wave [26] while in donor **7** the potentials of the successive electron transfers are close enough that the c.v. gives the appearance of a single reversible peak, but has a slight shoulder [24]. Molecules **9** (*E*_1/2_ = −1.00 V vs SCE in DMF) [30–32] and **10** [33,35,37] extend the range of designs of neutral organic electron donors, although we are not aware of them being investigated as yet for the reduction of organic substrates.

In order to design both more potent electron donors, and donors with calibrated and targeted properties, the factors that drive the electron transfer(s) need to be clearly understood, and this paper now probes two factors that could impact on that.

## Results and Discussion

**Figure 2:**
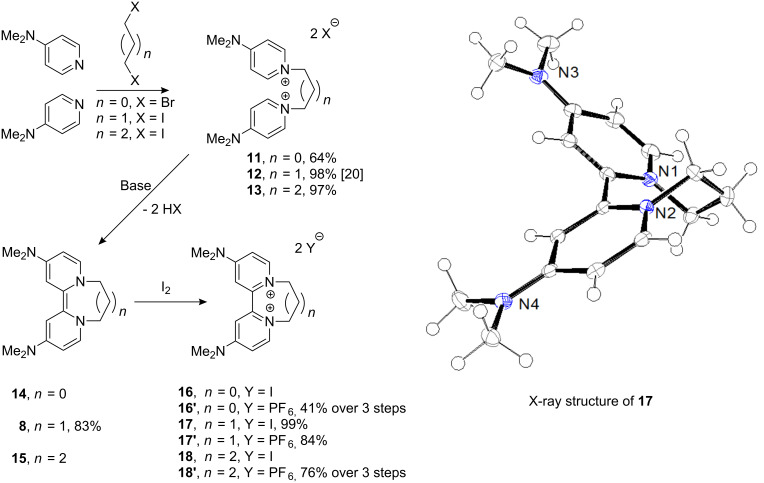
Formation of donors and oxidation to form diiodide salts, together with the ORTEP diagram of diiodide salt **17** (only the cation is shown).

Donor **8** has a number of attractive features. It is simply prepared from the reaction of 4-DMAP with 1,3-diiodopropane, followed by treatment of the product with base [26–28]. A wide range of analogues of 4-DMAP, which have been well studied in acylation chemistry [42–43], is already available. This suggests that preparation of analogues of **8** should also be straightforward. Hence, donor **8** was selected as the target for modification. The effect of modifying the length of a polymethylene chain linking the two pyridine-derived rings and the nature of the substituents on the 4- and 4′-positions of those pyridine rings were the points of particular interest. TDAE, **5**, has been used extensively as a two-electron transfer reagent, and many salts that feature its dication have been analysed by X-ray crystallography [44]. In these dications, the two ends of the molecule are twisted extensively to minimize interaction between the two positive charges. It is tempting to think that the degree of twist is linked to the power of the reducing agent. If twisting was not possible, then the driving force for removal of the second electron, for the conversion of the radical cation to the dication, should be diminished. To see if the same twist occurs with our donor **8**, the crystal structure of the disalt **17** was determined [twist (N–C–C–N = 52.5(3) degrees] (Figure 2). The degree of twist is limited by the three-carbon chain – a longer chain should afford greater flexibility and might afford a stronger donor, mirroring the findings of Ames et al. with a different series of compounds [18–20,22]. In contrast, shortening the polymethylene chain as in **14** should constrain rotation of the pyridine rings in the dication **16**, and hence make formation of **16** more difficult. To determine the effect of bridge-length on redox potential, the analogous donors **14** and **15** were prepared in situ and converted to their respective oxidized salts **16** and **18**, as shown in Figure 2, by reaction with iodine. Anion exchange to afford the corresponding hexafluorophosphate salts **16′** and **18′** was then carried out prior to cyclic voltammetry. (The iodide anions were exchanged since iodide ions would be electrochemically active, albeit at more positive potentials than feature in our studies.)

Cyclic voltammetry studies were carried out by adding deoxygenated solutions of the oxidized disalts **16′**–**18′** (rather than the electron donors) to the electrochemical cell and then carrying out the electrochemistry under an inert gas. The donors themselves are highly sensitive to traces of oxygen, and so are less convenient to weigh out than the disalts. All of the cyclic voltammograms showed reversible redox chemistry, featuring the transfer of two electrons, as indicated by calibration with ferrocene/ferrocenium (Fc/Fc^+^).

**Figure 3:**
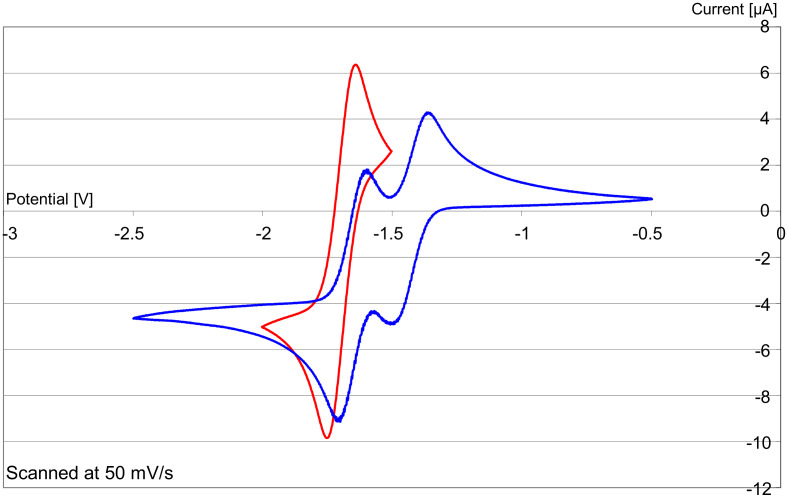
Cyclic voltammograms vs Fc/Fc^+^ of **17′** ↔ **8** (red) and **16′** ↔ **14** (blue).

Restricting the bridge length to two carbons made donor **14** a less effective reducing agent (Figure 3) compared to **8**, underlining the importance of flexibility of the inter-ring bond. Here, donor **8** shows a single two-electron wave [*E*^1^_1/2_ (DMF) = −1.24 V vs SCE (calibrated using Fc/Fc^+^)], while donor **14** shows two one-electron waves [*E*^1^_1/2_ (DMF) = −1.21 V, and *E*^2^_1/2_ (DMF) = −0.98 V vs SCE (calibrated using Fc/Fc^+^)]. The potential for loss of the first electron is similar in both compounds; however, the loss of the second electron from **8** is about 300 mV more negative than from **14**. This indicates a greater driving force for loss of the second electron in **8** than in **14**, consistent with the predicted difficulty in forming **14** as an essentially planar dication, where repulsion between the two charges would be more severe.

**Figure 4:**
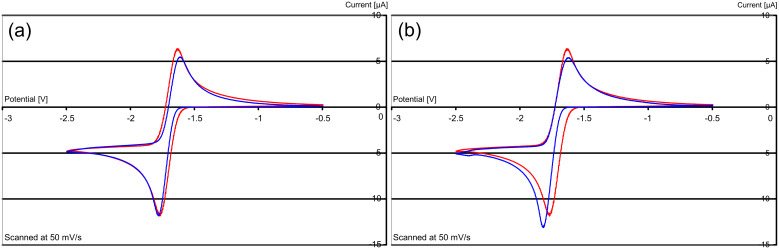
(a) Cyclic voltammograms vs. Fc/Fc^+^ of **17′** ↔ **8** (red) and **18′** ↔ **15** (blue) and (b) of **17′** ↔ **8** (red) and **20′** ↔ **19** (blue).

**Figure 5:**
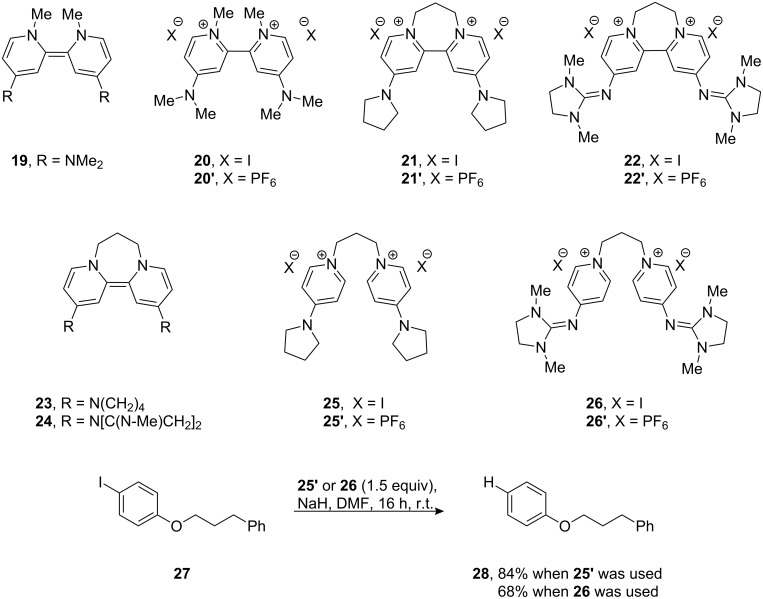
Electron donors, their oxidized dications and their reactions with **27**.

In contrast, comparison of the cyclic voltammograms of **18′** and **17′** (Figure 4a) showed only minor differences, with both showing a single wave corresponding to a two-electron reversible process at essentially the same potential (within 10 mV), so the increased flexibility does not benefit the two-electron donor **15** relative to **8**. Taking the idea of flexible rotation between the two halves of the molecule to its limit, we prepared compound **20′** [27] (Figure 5) and determined its cyclic voltammetric behaviour. As shown in Figure 4b, this [*E*_1/2_ (DMF) = −1.27 V vs SCE (calibrated using Fc/Fc^+^)] shows little difference from that of **17′**. [*E*_1/2_ (DMF) = −1.24 V vs SCE (calibrated using Fc/Fc^+^)]. Accordingly, permitting a freer rotation than seen in **17′** by extending the tether between the two pyridine-derived rings does not lead to enhanced donor properties.

**Figure 6:**
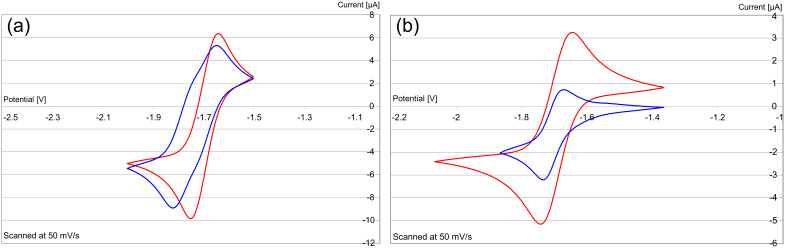
Cyclic voltammograms vs Fc/Fc^+^ (a) of **17′** ↔ **8** (red) and **21′** ↔ **23** (blue) and (b) of **17′** ↔ **8** (red) and **22′** ↔ **24** (blue) (at half the concn used for **17′**).

The other site of relatively easy variation in **8** was the dialkylamino group. 4-Pyrrolidinopyridine and 4-guanidinopyridine are significantly better catalysts [42–43] in acylation reactions than 4-DMAP. Their effectiveness depends on the delocalization of the electron pair on the 4-substituent into the pyridine ring. Accordingly, the disalts **21** and **22** were prepared from these 4-substituted pyridines [43,45] and converted into their hexafluorophosphates **21′** and **22′**, and then examined by cyclic voltammetry. Each showed a reversible two-electron redox wave (Figure 6). Redox equilibria related to **21** showed that the corresponding donor **23** was a stronger donor than donor **8** by about 90 mV for the transfer of its first electron, while the second electron occurs at the same potential as seen for donor **8**, while **24** transferred both of its electrons at the same potential and was within 10mV of **17′**.

The reactivity of these two donors was also investigated with substrate **27**. Here, **23** and **24** were prepared in situ from **25′** and **26**. This afforded the reductive de-iodination product **28** in good yield (84% when using **25′**, and 68% when using **26**).

These results are in accord with the previous reactions of donor **8**, and show that significantly more powerful donors than **8** cannot be attained simply by altering the tether length between the two pyridine units. Similarly, simple modifications to the 4′-substituent do not lead to very large changes in the redox properties of **8**. [The oxidation potentials of these new donors and the preceding examples mentioned in this paper are tabulated below in Table 1.] These outcomes are already helping our design of new, versatile and more powerful organic electron donors.

**Table 1:** Oxidation potentials of organic electron donors^a^

Electron Donor	*E*^1^_1/2_	*E*^2^_1/2_	Structure

**1**^b^	0.37 V	0.67 V	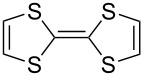
**4**^c^	−0.38 V(irr)		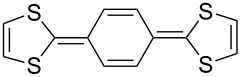
**5**^c^	−0.78 V	−0.61 V	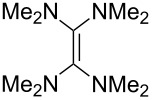
**6**	−0.82 V	−0.76 V	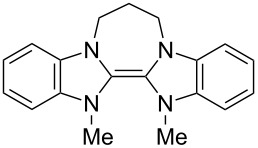
**7**	−1.20 V^e^		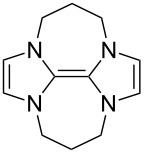
**8**	−1.24 V^e^		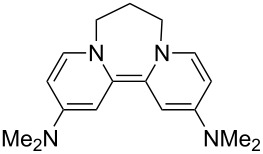
**9**	−1.00 V^d^		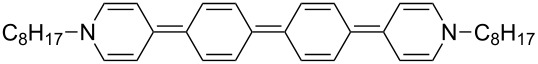
**10**	−0.32 V^e^		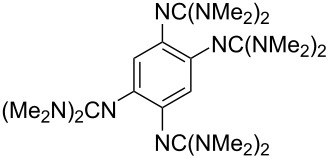
**14**	−1.21 V	−0.98 V	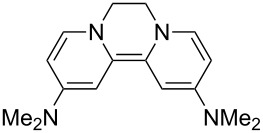
**15**	−1.23 V^e^		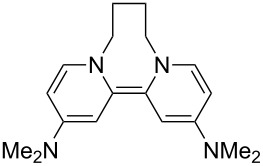
**19**	−1.27 V^e^		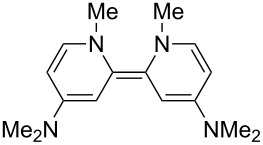
**23**	−1.33 V	−1.24 V	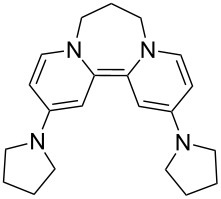
**24**	−1.24 V^e^		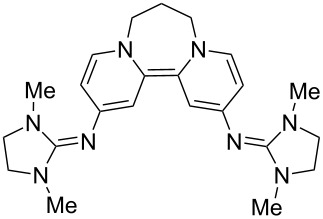

^a^All numbers have been converted for comparison with SCE; all experiments recorded in DMF, except where otherwise stated. ^b^Recorded in DCM. ^c^Recorded in MeCN. ^d^Recorded in THF. ^e^Two-electron wave.

## Supporting Information

Supporting Information features detailed information on experimental procedures and compound characterisation.

File 1 Experimental Part
