# Supplementary material for: Structure and reactivity in neutral organic electron donors derived from 4-dimethylaminopyridine
Source: Beilstein J Org Chem. 2010 Jul 5;6:73. doi: 10.3762/bjoc.6.73 (PMC2919259; doi:10.3762/bjoc.6.73)
Supplement: File 1 — Experimental Part [file Beilstein_J_Org_Chem-06-73-s001.pdf]

Supporting Information

for

Structure and reactivity in neutral organic electron donors derived from 4-dimethylaminopyridine

Jean Garnier[1], Alan R. Kennedy[1], Leonard E. A. Berlouis[1], Andrew T. Turner[2] and John A. Murphy*[1]

Address: [1]WestCHEM, Department of Pure and Applied Chemistry, University of Strathclyde, 295, Cathedral Street, Glasgow G1 1XL, U. K. and [2]PR&D Laboratory Building, AstraZeneca, Silk Road Business Park, Charterway, Macclesfield SK10 2NA, United Kingdom.

Email: Jean Garnier - jeangarn@hotmail.com; Alan A. Kennedy - a.r.kennedy@strath.ac.uk; Leonard E. A. Berlouis - l.berlouis@strath.ac.uk; Andrew T. Turner - andrew.t.turner@astrazeneca.com: John A. Murphy* - john.murphy@strath.ac.uk

* Corresponding author

*1,2-*bis*(*N',N'*-dimethyl-4-aminopyridinium)ethane dibromide (11)*

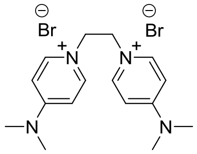

11

A solution of 1,2-dibromoethane (2.82 g, 1.3 ml, 15 mmol, 1.0 eq.) and 4-dimethylaminopyridine (4.58 g, 37.5 mmol, 2.5 eq.) in acetonitrile (100 ml) was stirred at reflux for 72 h, under an argon atmosphere. After cooling, diethyl ether (20 ml) was added, and the resulting solid filtered, washed with diethyl ether (3 x 100 ml) and dried under vacuum to give 1,2-*bis*(*N',N'*-dimethyl-4-aminopyridinium)ethane dibromide **11** [1] as a white powder (4.16 g, 64 %); mp: 316 °C (dec.) (lit. [2]) 308-310 °C); [Found: (ESI$^+$) (M-Br)$^+$ 351.1183. $C_{16}H_{24}(^{79}Br)_2N_4$ requires M-Br 351.1179]; ν_{max}(film)/cm^{-1} 3465, 3018, 1648, 1570, 1212, 1187; ^{1}H-NMR (400 MHz, DMSO-d$_6$) δ 3.20 (12H, s, N(CH_3)$_2$), 4.69 (4H, s, NCH$_2$), 7.08 (4H, d, *J* = 7.8 Hz, ArH), 8.22 (4H, d, *J* = 7.8 Hz, ArH); ^{13}C-NMR (100 MHz, DMSO-d$_6$) δ 39.8 (CH$_3$), 55.6 (CH$_2$), 107.8 (CH), 142.1 (CH), 155.9 (C); *m/z* (ESI$^+$) 353 [(M-Br)$^+$,^{81}Br , 2 %], 351 [(M-Br)$^+$,^{79}Br , 2 %], 231 (1), 229 (1), 149 (4), 136 (100), 123 (4).

1,2-**bis*****(*****N',N'*****-dimethyl-4-aminopyridinium)butane diiodide (13)***

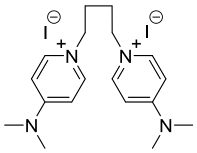

13

A solution of 1,4-diiodobutane (3.1 g, 10 mmol, 1.0 eq.) and 4-dimethylaminopyridine (3.05 g, 25 mmol, 2.5 eq.) in acetonitrile (50 ml) was stirred at reflux for 72 h, under an inert atmosphere. After cooling, diethyl ether (20 ml) was added, and the resulting solid filtered, washed with diethyl ether (3 x 100 ml) and dried under vacuum to give *1,2-*bis*(*N',N'*-dimethyl-4-aminopyridinium)butane diiodide* (**13**) as a white powder (5.39 g, 97 %); mp: 270-280 °C; [Found: (ESI$^+$) (M-I)$^+$ 427.1352. $C_{18}H_{28}I_2N_4$ requires M-I 427.1353]; ν_{max}(film)/cm^{-1} 3504, 3428, 3036, 1649, 1566, 1180, 828; ^{1}H-NMR (400 MHz, DMSO-d$_6$) δ 1.73 (4H, broad s, CH_2), 3.20 [12H, s, $N(CH_3)_2$], 4.21 (4H, broad s, NCH_2), 7.06 (4H, d, *J* = 7.5 Hz, ArH), 8.31 (4H, d, *J* = 7.5 Hz, ArH); ^{13}C-NMR (100 MHz, DMSO-d$_6$) δ 26.9 (CH_2), 39.8 (CH_3), 55.8 (CH_2), 107.7 (CH), 141.9 (CH), 188.8 (C) ; *m/z* (ESI$^+$) 427 [(M-I)$^+$, 8 %], 150 (100).

Preparation of hexafluorophosphate salts for cyclic voltammetry.

(a) ***General cyclization method for the preparation of electron donors (e.g. 8, 14,15) in situ and oxidation to their diiodide salts (e.g 16 – 18).***

Under an inert atmosphere (nitrogen), NaH (10 eq.) was added to a stirred solution of the disalt precursor (1 eq.) in degassed DMF (5 ml / mmol of disalt), and left to react at r.t. (for 3 h if not stated otherwise). Filtration of the excess sodium hydride/sodium

iodide/sodium bromide salts produced a solution of the desired electron donor, which was poured into a solution of iodine (1.2 eq.) in diethyl ether (20 ml / mmol of disalt). Addition of excess diethyl ether (20 ml / mmol disalt) followed by filtration of the resulting solid and drying under vacuum gave the crude diodide salt.

(b) ***General method for the conversion of diiodide salts to dihexafluorophosphate salts***

A solution of the diiodide salt obtained as above in a 50/50 mixture of water/methanol (2ml / mmol dication) was treated with $NaPF_6$ (2.5 eq.) in water (3-4 ml). The mixture was heated under reflux and water added dropwise until precipitation started. Methanol was then added dropwise until the precipitate redissolved. Slow cooling to r.t. led to crystallization. Filtration followed by drying at high temperature (100 °C) under vacuum gave the dihexafluorophosphate salt.

Preparation of N,N,N',N'-tetramethyl-6,7-dihydrodipyrido[1,2-a;2',1'-c]pyrazinediium -2,11-diamine dihexafluorophosphate (16')

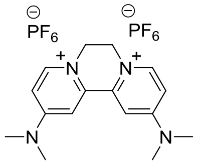

16'

Application of the general procedure for preparation of diiodide salts to 1,2-*bis*(*N',N'*-dimethyl-4-aminopyridinium)ethane dibromide **11** (864 mg, 2.00 mmol, 1.0 eq.) gave the corresponding crude diiodide salt (**16**) as a brown powder. Anion exchange of a

sample of the crude diiodide product (524 mg) according to the above general method afforded *N,N,N',N'-tetramethyl-6,7-dihydrodipyrido[1,2-a;2',1'-c]pyrazine-diium-2,11-diaminedihexafluorophosphate* (**16'**) as a light brown powder [241 mg, 41 %]; mp: 250-255 °C (dec.); [Found: (ESI$^+$) (M-PF$_6$)$^+$ 415.1489. $C_{16}H_{22}F_{12}N_4P_2$ requires M-PF$_6$ 415.1481]; ν_{max}(film)/cm^{-1} 3125, 2948, 1642, 1570, 836, 558; ^{1}H-NMR (400 MHz, DMSO-d$_6$) δ 2.30 (6H, s, NCH$_3$), 3.42 (6H, s, NCH$_3$), 4.65 (4H, s, NCH$_2$), 7.23 (2H, dd, *J* = 7.6, 3.0 Hz, ArH), 7.74 (2H, d, *J* = 3.0 Hz, ArH), 8.43 (2H, d, *J* = 7.6 Hz, ArH); ^{13}C-NMR (100 MHz, DMSO-d$_6$) δ 40.1 (CH$_3$), 49.1 (CH$_2$), 107.4 (CH), 107.6 (CH), 138.3 (C), 143.1 (CH), 156.1 (C); *m/z* (ESI$^+$) 415 [(M-PF$_6$)$^+$, 6 %], 241 (21), 135 (100).

Preparation of N,N,N',N'*-tetramethyl-7,8-dihydro-6*H*-dipyrido[1,2a-2',1'c]-[1,4]-diazepinium-2,12-diamine diiodide (17)*

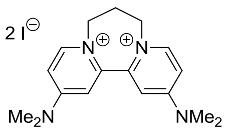

17

N,N,N',N'-tetramethyl-7,8-dihydro-6*H*-dipyrido[1,2-a;20,10-c][1,4]diazepine-2,12-diamine (**8**) [3] (170 mg, 0.6 mmol, 1.0 eq.) was dissolved in dry and degassed acetonitrile (5 ml) under a nitrogen atmosphere, and transferred into a stirred solution of iodine (254 mg, 1 mmol, 1.7 eq.) in diethyl ether (10 ml). After 30 min, the mixture was filtered, and the solid washed thoroughly with diethyl ether. Drying under vacuum gave *N,N,N',N'-tetramethyl-7,8-dihydro-6H-dipyrido[1,2a-2,1c]-[1,4]-diazepinium-2,12-diamine diiodide* (**17**) (317 mg, 99 %) as a light brown powder.; mp: 182-186 °C (dec);

[Found: (ESI$^+$) (M-I)$^+$ 411.1043. $C_{17}H_{24}I_2N_4$ requires M-I, 411.1040]; ν_{max}(KBr)/cm^{-1} 2924, 1640, 1573, 1531, 1399; ^{1}H-NMR (400 MHz, DMSO-d$_6$) δ 2.40-2.43 (2H, m, CH_2), 3.30 (6H, s, NCH_3), 3.34 (6H, s, NCH_3), 3.92-4.00 (2H, m, NC*H*HCH$_2$CH*H*N), 4.53-4.57 (2H, m, NCH*H*CH$_2$C*H*HN), 7.34 (2H, dd, *J* = 7.6, 3.1 Hz, ArH), 7.40 (2H, d, *J* = 3.1 Hz, ArH), 8.44 (2H, d, *J* = 7.6 Hz, ArH); ^{13}C-NMR (100 MHz, DMSO-d$_6$) δ 28.6 (CH_2), 40.3 (CH_3), 40.5 (CH_3), 51.0 (CH_2), 108.1 (CH), 111.8 (CH), 142.7 (C), 143.3 (CH), 156.2 (C); *m/z* (ESI$^+$) 411 [(M-I)$^+$, 8 %], 229 (8), 383 (12), 243 (55), 227 (100), 200 (15), 179 (30), 163 (35), 142 (20), 134 (80), 112 (70).

The structure of this sample was determined by X-ray crystallography.

Crystal data for **17**: $C_{17}H_{24}I_2N_4$, M_r = 538.20, triclinic, space group P $\bar{1}$, a = 8.1751(2), b= 10.4009(2), c= 13.5326(3) Å, α= 106.884(1), β= 96.752(1), γ= 110.616(1) °, V = 999.02(4) Å^3, Z = 2, λ = 0.71073 Å, μ = 3.153 mm^{-1}, T = 123 K; 22742 reflections, 4585 unique, R_{int} 0.0344; final refinement to convergence on F^2 gave R = 0.0240 (F, 4081 obs. data only) and R_w = 0.0546 (F^2, all data), GOF = 1.070. Full details have been deposited as a cif file with the CCDC. They are available on request from http://www.ccdc.cam.ac.uk quoting the depository number CCDC 774828.

Preparation of N,N,N',N'-tetramethyl-7,8-dihydro-6H-dipyrido[1,2a-2',1'c]-[1,4]-diazepinium-2,12-diamine dihexafluorophosphate (17')

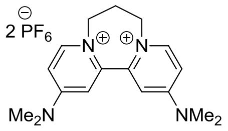

17'

Application of the general procedure for diiodide salt preparation to 1,3-*bis*(*N',N'*-dimethyl-4-aminopyridinium)propane diiodide (**12**) (2.16 g, 4 mmol, 1.0 eq.) gave the corresponding crude diiodide salt **17** as a brown powder. The general procedure for conversion to the dihexafluorophosphate salt was used to obtain *N,N,N',N'-tetramethyl-7,8-dihydro-6H-dipyrido[1,2a-2,1c]-[1,4]-diazepinium-2,12-diamine dihexafluorophosphate* (**17'**) as pale yellow needles (1.92 g, 84 %); mp: 282-287 °C (dec.); [Found: (ESI$^+$) (M-PF$_6$)$^+$ 429.1631. $C_{17}H_{24}F_{12}N_4P_2$ requires M-PF$_6$, 429.1637]; ν_{max}(film)/cm^{-1} 3118, 2948, 1644, 1572, 1536, 838, 557; ^{1}H-NMR (400 MHz, DMSO-d$_6$) δ 2.37-2.42 (2H, m, CH$_2$), 3.28 (6H, s, NCH$_3$), 3.31 (6H, s, NCH$_3$), 3.90-3.98 (2H, m, NC*H*HCH$_2$CH*H*N), 4.50-4.56 (2H, m, NCH*H*CH$_2$C*H*HN), 7.24 (2H, dd, *J* = 7.6, 3.1 Hz, ArH), 7.39 (2H, d, *J* = 3.1 Hz, ArH), 8.42 (2H, d, *J* = 7.6 Hz, ArH); ^{13}C-NMR (100 MHz, DMSO-d$_6$) δ 28.6 (CH$_2$), 40.1 (CH$_3$), 40.2 (CH$_3$), 51.0 (CH$_2$), 108.1 (CH), 111.5 (CH), 143.0 (C), 143.6 (CH), 156.3 (C); *m/z* (ESI$^+$) 429 [(M-PF$_6$)$^+$, 40 %], 142 (100).

Preparation of* N,N,N',N'*-tetramethyl-6,7,8,9-tetrahydrodipyrido[1,2-a;2',1'-c]-(1,4)-diazocinium-2,13-diamine dihexafluorophosphate (18')

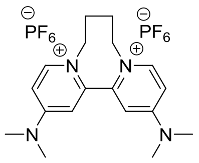

18'

Application of the general procedure for diiodide salt preparation to 1,2-*bis*(*N',N'*-dimethyl-4-aminopyridinium)butane diiodide **13** (1.11 g, 2.00 mmol, 1.0 eq.) gave the corresponding crude diiodide **18** as a yellow powder. Anion exchange according to the general method afforded *N,N,N',N'-tetramethyl-6,7,8,9-tetrahydrodipyrido[1,2-a;2',1'-c]-(1,4)-diazocinium-2,13-diamine dihexafluoro-phosphate* (**18'**) as a light yellow powder (889 mg, 76 %); mp: 268-275 °C (dec.); [Found: (ESI$^+$) (M-PF$_6$)$^+$ 443.1797. $C_{18}H_{26}F_{12}N_4P_2$ requires M-PF$_6$ 443.1794]; ν_{max}(film)/cm^{-1} 3103, 2957, 1643, 1579, 1537, 1171, 836, 558; ^{1}H-NMR (400 MHz, DMSO-d$_6$) δ 1.73-1.85 [2H, m, NCH$_2$(CH*H*)], 2.07-2.11 [2H, m, NCH$_2$(C*H*H)], 3.67-3.73 (2H, m, NC*H*H), 4.37-4.42 (2H, m, NCH*H*), 7.25 (2H, d, *J* = 3.1 Hz, ArH), 7.31 (2H, dd, *J* = 7.7, 3.1 Hz, ArH), 8.53 (2H, d, *J* = 7.7 Hz, ArH); ^{13}C-NMR (100 MHz, DMSO-d$_6$) δ 27.3 (CH$_2$), 40.0 (CH$_3$), 53.8 (CH$_2$), 109.3 (CH), 111.0 (CH), 142.9 (C), 143.5 (CH), 155.9 (C); *m/z* (ESI$^+$) 443 [(M-PF$_6$)$^+$, 5 %], 297 (35), 243 (38), 149 (100).

Preparation of 26.

***First step: 4-(1,3-dimethylimidazolidin-2-ylidene)aminopyridine)* A** [4].

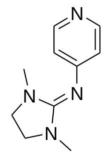

A

A stirred solution of 4-aminopyridine (1.88 g, 20 mmol, 1.0 eq.) and triethylamine (2.02 g, 2.8 ml, 20 mmol, 1.0 eq.) in acetonitrile (15 ml) was cooled to 0 °C under an argon atmosphere and a solution of 2-chloro-1,3-dimethylimidazolidium chloride [5] (3.38 g, 20 mmol, 1.0 eq.) in acetonitrile (45 ml) was added dropwise to produce a precipitate. The mixture was then heated under reflux overnight. After the addition of sodium hydroxide (800 mg, 20 mmol, 1.0 eq.) the reaction mixture was stirred for 1 h, and the solvent evaporated. Potassium hydroxide solution (50 %, 20 ml) was added and the mixture extracted with acetonitrile (4 x 20 ml). The organic phase was dried with a mixture of sodium sulfate and charcoal, and filtered through Celite. Evaporation of the solvent provided *4-(1,3-dimethylimidazolidin-2-ylidene)aminopyridine* A as a waxy white solid (3.32 g, 87 %); ν_{max}(film)/cm^{-1}; 2947, 2877, 1620, 1574, 1496, 1417, 1289; [Found: (ESI^+) $(M+H)^+$ 191.1292. $C_{10}H_{14}N_4$ requires M+H, 191.1291]; ^{1}H-NMR (400 MHz, $CDCl_3$) δ 2.61 (6H, s, NCH_3), 3.29 (4H, s, NCH_2), 6.60-6.62 (2H, m, ArH), 8.15-8.16 (2H, m, ArH); ^{13}C-NMR (100 MHz, $CDCl_3$) δ 35.1 (CH_3), 48.3 (CH_2), 117.5 (CH), 149.7 (CH), 157.4 (C); *m/z* (ESI^+) 191 [$(M+H)^+$, 100 %].

Preparation of 1,3-*bis*[4-(1,3-dimethylimidazolidin-2-ylidene]aminopyridinium)propane diiodide (26)

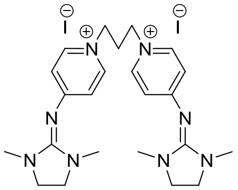

26

A stirred solution of 1,3-diiodopropane (947 mg, 0.370 ml, 3.2 mmol, 1.0 eq.) and 4-(1,3-dimethylimidazolidin-2-ylidene)aminopyridine (1.53 g, 8 mmol, 2.5 eq.) in anhydrous acetonitrile (25 ml) was refluxed under an argon atmosphere for 72 h. After cooling, anhydrous diethyl ether (20-30 ml) added in small portions over a week to initiate slow crystallisation. Filtration followed by washing with more anhydrous diethyl ether (100 ml) and evaporation of the remaining solvents under vacuum gave *1,3-bis(4-(1,3-dimethylimidazolidin-2-ylidene)aminopyridine) propane diiodide* (**26**) as large hygroscopic cubes (1.99 g, 92 %); mp: 268-272 °C; [Found: (ESI^+) $(M-I)^+$ 549.1939. $C_{23}H_{34}I_2N_8$ requires M-I, 549.1946]; ^{1}H-NMR (400 MHz, DMSO-d_6) δ 2.31 (2H, quintet, J = 7.2 Hz, $NCH_2CH_2CH_2N$), 2.72 (12H, s, NCH_3), 3.65 [8H, s, $N(CH_2)_2N$], 4.14 (4H, t, J = 7.2 Hz, $NCH_2CH_2CH_2N$), 6.61 (4H, d, J = 7.5 Hz, ArH), 8.05 (4H, d, J = 7.5 Hz, ArH); ^{13}C-NMR (100 MHz, DMSO-d_6) δ 31.0 (CH_2), 33.1 (CH_3), 47.3 (CH_2), 53.7 (CH_2), 114.6 (CH), 141.5 (CH), 160.9 (C), 162.5 (C); *m/z* (ESI^+) 549 [$(M-I)^+$, 10 %], 211 (100).

Preparation of* N,N'*-bis(1,3-dimethylimidazolidin-2-ylidene)-7,8-dihydro-6*H*-dipyrido [1,2-a,2',1'-c]-[1,4]-diazepinium-2,12-diamine dihexafluorophosphate (22')

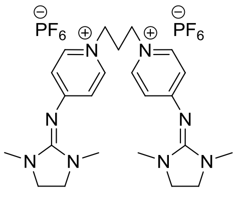

22'

Under a nitrogen atmosphere, KHMDS in toluene (0.880 ml, 0.5 M, 0.44 mmol, 2.2 eq.) was added to a stirred solution of 1,3-*bis*(4-(1,3-dimethylimidazolidin-2-ylidene)aminopyridine)-propane diiodide **22** (132 mg, 0.2 mmol, 1.0 eq.) in degassed DMF (5 ml), and left to react at r.t. for 3 h. The reaction mixture was then poured into a solution of iodine (61 mg, 0.24 mmol, 1.2 eq.) in diethyl ether (25 ml). Addition of excess diethyl ether (20 ml) was followed by filtration of the crude product. Application of the general procedure for dication purification to the crude product provided *N,N'-bis(1,3-dimethylimidazolidin-2-ylidene)-7,8-dihydro-6H-dipyrido[1,2-a;2',1'-c]-[1,4]-diazepinium-2,12-diamine dihexafluorophosphate* **22'** as a light brown powder (75 mg, 54 %); mp: 178-180 °C (dec.); ν_{max}(film)/cm^{-1} 2943, 1642, 1578, 1373, 1194, 1029, 837, 557; [Found: (ESI$^+$) (M-2 PF_6)$^{2+}$ 210.1367. $C_{23}H_{32}F_{12}N_8P_2$ requires (M-2 PF_6)/2 , 210.1369]; ^{1}H-NMR (400 MHz, DMSO-d$_6$) δ 2.29-2.35 (2H, m, $NCH_2CH_2CH_2N$), 2.78 (12H, s, NCH_3), 3.69 (8H, s, NCH_2CH_2N), 3.88-3.96 (2H, m, NC*H*HCH$_2$C*H*HN), 4.38-4.42 (2H, m, NCH*H*CH$_2$CH*H*N), 6.71 (2H, dd, *J* = 7.2, 2.4 Hz, ArH), 6.86 (2H, d, *J* = 2.4 Hz, ArH), 8.12 (2H, d, *J* = 7.2 Hz, ArH); ^{13}C-NMR (100 MHz, DMSO-d$_6$) δ 28.6 (CH_2), 33.0 (CH_3), 47.4 (CH_2), 51.8 (CH_2), 114.2 (CH), 117.4 (CH),

143.1 (CH), 143.3 (C), 161.2 (C), 162.5 (C); *m/z* (ESI^+) 565 [$(M-PF_6)^+$, 15 %], 210 (100). This disalt was used in cyclic voltammetry experiments.

Cyclic voltammetry

General procedure. Cyclic voltammetry was performed with a standard three-electrode system, controlled by an Autolab™ potentiostat/galvanostat PGSTA30. The auxiliary electrode was a platinum wire, the working electrode a platinum disc 0.7 mm in diameter, polished before each session. Finally, the Ag/AgCl reference was a Thermo™ electrode, with double compartment, with saturated KCl solution in the inner cell and the organic electrolyte in the double-junction compartment. Experiments were conducted under an inert atmosphere (nitrogen), in a glove box at room temperature (20-23 °C).

For each session of measurements, fresh electrolyte was prepared in degassed DMF using TBAHFP as the supporting salt (0.1 M). Solutions of dication were prepared in 10 ml volumetric flasks. For calibration, the redox potential of ferrocene was measured at the beginning and at the end of every session, providing the average value used for determining $E_{1/2}$ *vs.* Fc/Fc^+ (published potential in similar conditions: 0.45 V *vs.* SCE [6]). Cyclic voltammetry was measured at a scan rate of 50 mV per second.

General One-Pot Reduction Procedure for Aryl Halides

Under an inert atmosphere, anhydrous DMF (5 mL) was added to a mixture of the aryl iodide (0.3 mmol, 1.0 equiv), donor precursor (0.45 mmol, 1.5 equiv of disalt) and washed NaH (108 mg, 15 mmol). Overnight stirring at r.t. followed by filtration, extraction (H_2O–Et_2O) and purification by column chromatography provided the reduced products as reported.

One-pot reduction of 27

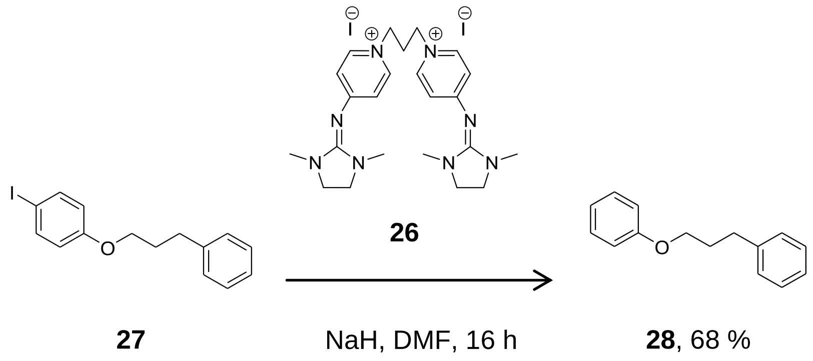

Application of the one-pot procedure to 1-iodo-4-(3-phenylpropoxy)benzene **27** (105 mg, 0.31 mmol, 1.0 eq.) using 1,3-*bis*(4-(1,3-dimethylimidazolidin-2-ylidene)aminopyridinium) propane diiodide **26** (304 mg, 0.45 mmol, 1.5 eq.) as the disalt precursor in an overnight reaction at r.t. provided (3-phenylpropoxy)benzene **28** as a colourless oil (45 mg, 68 %). [Found: (ESI$^+$) $(M+NH_4)^+$, 230.1538. $C_{15}H_{16}O$ requires $M+NH_4$, 230.1539]; ν_{max}(film)/cm^{-1} 3062, 3027, 2946, 2870, 1600, 1497, 1245, 1038, 751; ^{1}H-NMR (400 MHz, $CDCl_3$) δ 2.15 (2H, m, CH_2), 2.86 (2H, t, J = 7.5 Hz, $PhCH_2$), 4.01 (2H, t, J = 6.3 Hz, OCH_2), 6.93-7.00 (3H, m, ArH), 7.22-7.35 (7H, m, ArH); ^{13}C-NMR (100 MHz, $CDCl_3$) δ 31.1 (CH_2), 32.4 (CH_2), 67.0 (CH_2), 114.8 (CH),

120.8 (CH), 126.1 (CH), 128.6 (CH), 128.7 (CH), 129.6 (CH), 141.8 (C), 159.3 (C); *m/z* (CI^+) 230 [$(M+NH_4)^+$, 100 %], 212 (M^+, 20 %), 118 (10), 108 (13), 91 (22).

*Preparation of 1,3-*bis*(4-pyrrolidinopyridinium)propane dihexafluoro-phosphate (25')*

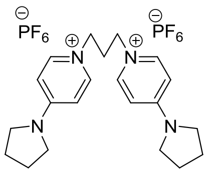

25'

A stirred solution of 1,3-diiodopropane (947 mg, 0.370 ml, 3.2 mmol, 1.0 eq.) and 4-pyrrolidinopyridine (1.41 g, 9.5 mmol, 2.11 eq.) in acetonitrile (20 ml) was refluxed under an argon atmosphere for 72 h. After addition of diethyl ether (20 ml), the precipitate was filtered, washed with more diethyl ether and finally dried under vacuum to yield a highly hygroscopic crude product. Application of the general cyclization procedure for preparation of diiodide salt and then the general procedure for conversion to the dihexafluorophosphate salt gave *1,3-bis(4-pyrrolidinopyridinium)propane dihexafluorophosphate* (***25'***) as 5 mm long needles (2.31 g, 82 %); mp: 216-220 °C; ν_{max}(film)/cm^{-1} 3104, 3073, 2969, 2879, 1653, 1574, 1196, 841; [Found: (ESI^+) $(M-PF_6)^+$ 483.2108. $C_{21}H_{30}F_{12}N_4P_2$ requires $M-PF_6$, 483.2107]; ^{1}H-NMR (400 MHz, DMSO-d_6) δ 2.0-2.03 (8H, m, $NCH_2CH_2CH_2CH_2N$), 2.32 (2H, quintet, *J* = 7.3 Hz, $NCH_2CH_2CH_2N$), 3.47-3.51 (8H, m, $NCH_2CH_2CH_2CH_2N$), 4.20 (4H, t, *J* = 7.3 Hz, $NCH_2CH_2CH_2N$), 6.89 (4H, d, *J* = 7.6 Hz, ArH), 8.22 (4H, d, *J* = 7.6 Hz, ArH); ^{13}C-NMR (100 MHz, DMSO-d_6) δ 24.7 (CH_2),

31.0 (CH_2), 48.3 (CH_2), 53.9 (CH_2), 108.3 (CH), 141.9 (CH), 153.0 (C); *m/z* (ESI^+) 483 [$(M-PF_6)^+$, 85 %], 169 (100).

*Preparation of 2,12-dipyrrolidino-7,8-dihydro-6*H*-dipyrido[1,2-a;2',1'-c]-[1,4]-diazepinium dihexafluorophosphate (21')*

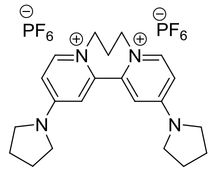

21'

Application of the general cyclization procedure for preparation of diiodide salt **25** (628 mg, 1 mmol, 1.0 eq.) and then the general procedure for conversion to the dihexafluorophosphate salt provided *2,12-dipyrrolidino-7,8-dihydro-6*H*-dipyrido[1,2-a;2',1'-c]-[1,4]-diazepinium dihexafluorophosphate* (**21'**) as long needles (531 mg, 85 %); mp: 260-265 °C; ν_{max}(film)/cm^{-1} 2972, 2882, 1651, 1578, 1199, 839; [Found: (ESI^+) $(M-PF_6)^+$ 481.1953. $C_{21}H_{28}F_{12}N_4P_2$ requires $M-PF_6$, 481.1950]; ^{1}H-NMR (400 MHz, DMSO-d_6) δ 2.00-2.10 (8H, m, $NCH_2CH_2CH_2CH_2N$), 2.35-2.45 (2H, m, $NCH_2CH_2CH_2N$), 3.45-3.55 (2H, m, $NCH_2CH_2CH_2CHHN$), 3.56-3.62 (4H, m, $NCH_2CH_2CH_2CH_2N$), 3.70-3.80 (2H, m, $NCH_2CH_2CH_2CHHN$), 3.91-3.99 (2H, m, $NCHHCH_2CHHN$), 4.50-4.55 (2H, m, $NCHHCH_2CHHN$), 7.09 (2H, dd, *J* = 7.4, 2.8 Hz, ArH), 7.25 (2H, d, *J* = 2.8 Hz, ArH), 8.41 (2H, d, *J* = 7.4 Hz, ArH); ^{13}C-NMR (100 MHz, DMSO-d_6) δ 24.6 (CH_2), 24.7 (CH_2), 28.7 (CH_2), 48.7 (CH_2), 48.8 (CH_2), 51.1 (CH_2), 108.7 (CH), 112.0 (CH), 142.5 (CH), 143.5 (C), 153.5 (C); *m/z* (ESI^+) 481 [$(M-PF_6)^+$, 100 %], 168 (54). This disalt was used in cyclic voltammetry experiments.

One-pot reduction of (27) using (25').

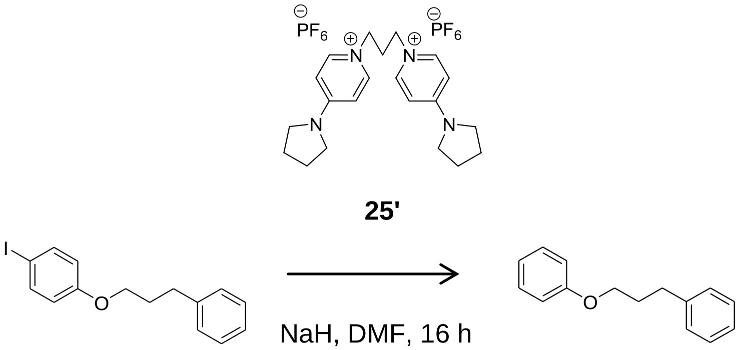

25'

NaH, DMF, 16 h

27 **28,** 84 %

Application of the one-pot reduction procedure to 1-iodo-4-(3-phenylpropoxy)benzene **27** (100 mg, 0.30 mmol, 1.0 eq.) with 1,3-*bis*(4-pyrrolidinopyridinium)propane dihexafluorophosphate (**25'**) (282 mg, 0.45 mmol, 1.5 eq.) as the disalt precursor in an overnight reaction at r.t. provided (3-phenylpropoxy)benzene (**28**) as a colourless oil (53 mg, 84 %). Data for **28** agreed with those reported above.

References

[1]. Katritzky, A. R.; Mokrosz, M. J. *Heterocycles* **1984,** *22*, 505.

[2]. Sarkunam, N.; Nallu, M. *J. Indian Chem. Soc.* **2004,** *81*, 978.

[3]. Murphy, J. A.; Garnier, J.; Park, S. R.; Schoenebeck, F.; Zhou, S. Z.; Turner, A. T. *Org. Lett.* **2008,** *10*, 1227. doi:10.1021/ol800134g

[4]. Herres-Pawlis, S.; Neuba, A.; Seewald, O.; Seshadri, T.; Egold, H.; Florke,U.; Henkel, G. *Eur. J. Org. Chem.* **2005,** *22*, 4879. doi:10.1002/ejoc.200500340

[5]. We thank Dr. S. Z. Zhou for providing precursors.

[6]. Connelly, N. G.; Geiger, W. E. *Chem. Rev*. **1996**, *96*, 877. doi:10.1021/cr940053x
